# Supplementary material for: Preference classes in society for coastal marine protected areas
Source: PeerJ. 2019 Apr 23;7:e6672. doi: 10.7717/peerj.6672 (PMC6485209; doi:10.7717/peerj.6672)
Supplement: Supplemental Information 1 — Questionnaire used for the collection of data on trains during the study period. All questions are included. [file peerj-07-6672-s001.pdf]

MARINE PROTECTED AREAS IN WALES

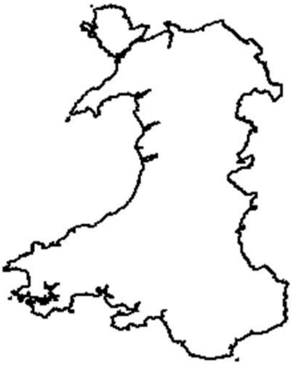

Bangor University is undertaking a survey to understand public views and preferences on Marine Protected Areas in Welsh waters.

YOUR OPINION IS IMPORTANT!!

The only way for decision-makers to understand people's points of view on protected areas is to undertake surveys like this.

The survey should take about 15 minutes to complete. All responses remain anonymous and confidential.

Thank you very much for taking part in this survey!

1. Marine Protected Areas in Wales

This survey asks for <u>your views</u> on having Marine Protected Areas (**MPAs**) on the Welsh coast to protect the variety of marine life and the sea floor.

- Marine Protected Areas (MPAs) are areas of the sea that are protected by law and where human activities are regulated for the protection of marine life.

Q1. Regarding your knowledge on Marine Protected Areas (MPAs), where would you place yourself on this diagram? (tick the most appropriate box)

Never heard of MPAs before			I consider I've got good knowledge on MPAs
→	→	→	→
☐	☐	☐	☐

2. General Information

The sea provides us with many services like fishing, recreation, wildlife watching and other uses.

- All these uses provided by the sea play a very important role on the economy and on people's wellbeing.
- The inadequate management of some of these activities has lead in many cases to the degradation of the seas.

Q2. Please answer each question by ticking the appropriate box.

	Yes	No
Is your job directly tied to the sea? (fishing, shipping, marine recreation, marina, etc.)	☐	☐
Do you or does anyone in your family fish commercially?	☐	☐

Q3. Have you done any of the following sea-related activities in the past 12 months? (please tick all that apply)

Sea-angling	☐	Surfing	☐
Diving	☐	Snorkelling	☐
Kayaking/Canoeing	☐	Sailing	☐
Marine wildlife watching (whales, dolphins, seals, seabirds…)	☐		
Other (please specify)	________________		

Q4. Do you live within 10 miles of the Welsh coast? ☐ Yes ☐ No

Q5. If your answer to the previous question was **NO**:

How many times have you visited the Welsh coast in the past 12 months?

Striking a balance between the uses of the sea and its conservation can be a complicated task.

- The sea is an important source of jobs and income, in Wales there are over 500 fishing boats; and 52,000 jobs are linked to the sea, mainly through tourism. In 2003, the Welsh fishing industry generated £79.6 million in revenues (0.1% of total revenues in the Welsh economy). Some of the activities however can sometimes damage habitats, pollute the waters or harvest too many fish.

- One way to resolve the conflict between economy and environmental protection is through the establishment of Marine Protected Areas (MPAs).

The establishment of an MPA does not mean the exclusion of all types of activities. MPAS can offer different levels of protection, possible levels of protection could be:

Level of protection
**Level 1: No-take zone**<br>All activities are prohibited within these areas<br>**Level 2: Education and Scientific research**<br>Only activities related to scientific research and education are allowed. These activities are regulated and only a certain amount of resources can be extracted for these purposes.<br>**Level 3: Non-extractive recreational activities**<br>Certain recreational activities like diving, sailing, surfing, kayaking, wildlife sighting are allowed, plus Level 2 uses. Activities are regulated.<br>**Level 4: Restricted fishing activities**<br>Recreational and commercial fishing using fishing equipment that does not damage the sea floor is allowed, plus Level 2 & 3 uses. These activities are tightly regulated

Q6. Please tick the one box that most closely represents your view. There are no right or wrong answers, your opinion is what's important.

MPAs provide a good way to get the right balance between conservation and activities such as fishing or shipping					
Completely True	Mostly True	Somewhat True	Slightly True	Not at all True	I am unsure
☐	☐	☐	☐	☐	☐

3. Marine Protected Areas. Benefits & Costs

Benefits

- The protection of marine life from damaging activities within an MPA results in an increase in the number and size of plants and animals living in the MPA.

- MPAs can protect entire habitats (i.e. from fish, to the creatures living on the seabed, to the seabed itself).

- Marine mammals, seabirds, fish and shellfish benefit from MPAs as these areas hold more food for them.

- Some of the benefits for society include:
  - Opportunities for nature-based recreation and tourism. MPAs with “no-take” zones are often major tourist attractions; they can be good way to raise the profile of an area for tourism and to broaden the local economic opportunities.

  - MPAs can play an important role in the recovery of commercial fish stocks. MPAs act as a refuge for fish. These fish can then move out of the MPA into surrounding waters increasing commercial and recreational catches.

  - Opportunities for education and scientific research about marine ecosystems

Q7. Please tick the one box that most closely represents your view for each of the questions.

	Completely True	Mostly True	Somewhat True	Slightly True	Not at all True	I am unsure
There are conservation benefits related to MPAs	☐	☐	☐	☐	☐	☐
There is no need for MPAs in Wales because the seas around the Welsh coasts are in good health	☐	☐	☐	☐	☐	☐

Costs

The establishment of MPAs also has costs for the users of the sea.

- The closure of areas of the seafloor to certain activities might increase costs for those commercial activities affected by the MPA.
- Increased production costs in turn might have an effect on consumer prices (i.e. seafood, energy).
- There are associated costs related to the management and enforcement of the MPA.
- Regulation of recreational, scientific and commercial activities allowed within the borders of the MPA might increase.

Q8. Please tick the one box that most closely represents your view for each of the questions.

	Completely True	Mostly True	Somewhat True	Slightly True	Not at all True	I am unsure
People who are affected by the creation of MPAs, like fishermen, should receive compensation for any financial losses derived from the establishment of MPAs	☐	☐	☐	☐	☐	☐
I'm willing to pay higher prices for sea-related products or services to preserve areas of the sea around Wales	☐	☐	☐	☐	☐	☐
Costs of MPAs will most likely be greater than the benefits obtained from them	☐	☐	☐	☐	☐	☐

4. Design and Size of Marine Protected Areas

The design and size of Marine Protected Areas (MPAs) is subject to much discussion.

- MPAs dedicated to conservation need to include a full representation of marine life, not only endangered species or those of commercial interest.
- Economic, social and political aspects need to be taken into account in the design of MPAs and they are as important as conservation aspects.
- Scientists agree that between 10% and 40% of regional seas might be needed to preserve all of the species of marine life in their natural habitats.

Q9. Please check the one box that most closely represents your opinion

	Completely True	Mostly True	Somewhat True	Slightly True	Not at all True	I am unsure
MPAs should be large enough to protect every type of organism living in the sea regardless of costs	☐	☐	☐	☐	☐	☐

5. Current Situation in Wales

- In Wales there are already some MPAs in place, approximately 30% of the sea lies within a type of MPA called a **S**pecial **A**rea of **C**onservation (**SAC**). A small area around Skomer Island is designated as **M**arine **N**ature **R**eserve (**MNR**) – see map-.

- These MPAs offer some protection to the habitats and the wildlife that live in them. Many activities (from commercial fishing to recreation) are still allowed although they are regulated to ensure the protection of the wildlife and habitats that sites were designated for.

- Currently, there are no MPAs in Wales that protect the variety of marine life from <u>all</u> damaging activities.

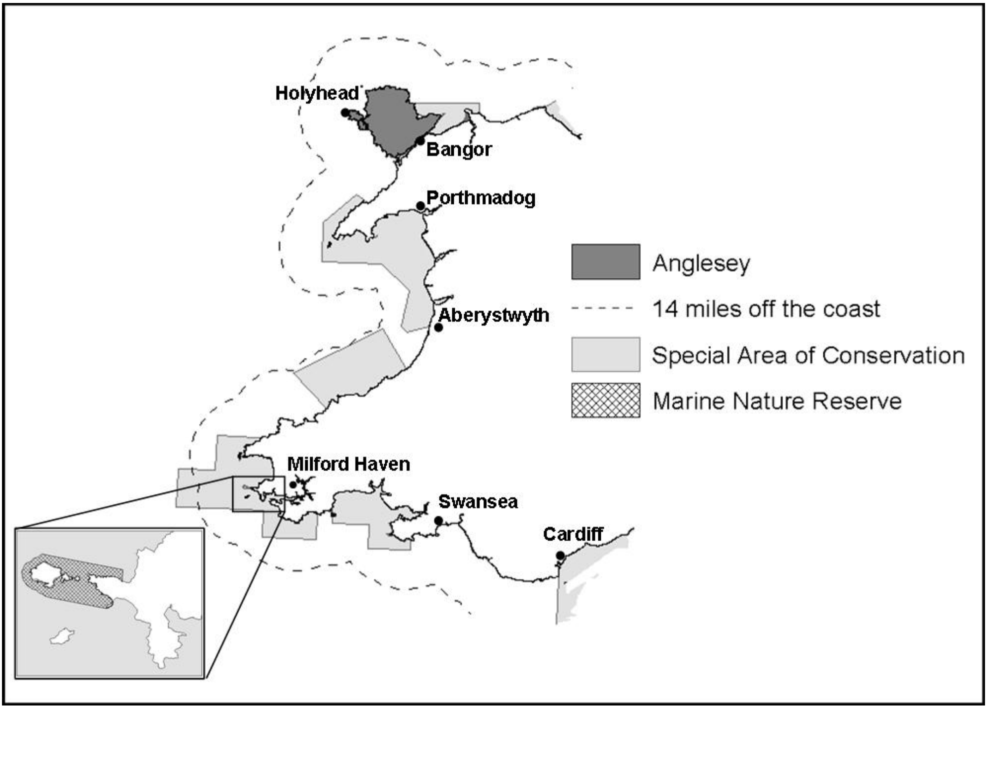

Future plans for Wales

The Welsh Assembly Government has committed to deliver a *network* of MPAs by 2012 to ensure the protection of the variety of marine life in Welsh waters.

- A *network* means that instead of having one big MPA, there would be a series of smaller MPAs distributed along the Welsh coast.
- The aim is to <u>complete</u> the existing network of MPAs by adding some new sites where the variety of marine life will be fully protected from all damaging activities.
- Most of these fully protected sites are likely to be located within existing Special Areas of Conservation.
- The network will be located in coastal waters (between the coast and 14 miles off the coast). -See map in previous page-

Some types of activities will be compatible with the conservation objectives of the MPA, so different levels of protection might be adopted for different areas.

Whether or not activities are allowed within Welsh MPAs depends upon the extent of damage or disturbance they are likely to cause.

Q10. For each of the statements below please tick the <u>one</u> box that most closely represents your opinion.

	Completely True	Mostly True	Somewhat True	Slightly True	Not at all True	I am unsure
The sea is a common resource and no one should be restricted from using it	☐	☐	☐	☐	☐	☐
There is no need to restrict uses that don't damage the seafloor in MPAs	☐	☐	☐	☐	☐	☐
Fishing equipment that sits on the seafloor and does not cause damage should be allowed in MPAs	☐	☐	☐	☐	☐	☐
Current levels of protection of the sea are enough	☐	☐	☐	☐	☐	☐
I like knowing that certain areas of the sea are being fully protected	☐	☐	☐	☐	☐	☐

6. Public Opinion on MPAs

- One of the main reasons for the failure of an MPA is the lack of users' agreement and support. It is extremely important to assess society's preferences in the design of MPAs.
- MPAs can be designed for different purposes: (1) specifically for the improvement of commercial fisheries; or (2) for the general conservation of the marine environment

- This survey concentrates on networks of MPAs designed for the protection of the variety of marine life and the sea floor (general conservation).

- Here we ask for your opinion on which type of MPAs you would like to see established in Wales. It does not matter if you are not a resident of Wales or if you don't visit the Welsh coast often, your opinion is still important.

- Over the next pages you are going to be presented with 5 imaginary situations (**Scenarios 1 to 5**). In each of them you will be asked to choose between 3 options (**Options A, B or Current Situation**). From these 3 options you will need to choose one. Consider each of the 5 scenarios independently from each other (e.g. if you are doing Scenario 1, concentrate only in the 3 options that appear there, do not pay attention to the rest of scenarios). The different scenarios will seem very similar but there are slight differences between them.

The following characteristics of the MPA will vary between scenarios:

- **Size of the MPA**: is the percentage of coastal waters that will be protected
- **Level of protection**: uses allowed within the boundaries of the MPA will vary
- **Cost**: to pay for the costs of establishing and managing MPAs you are asked to make a contribution each year to a neutral charity. The charity works with the government to negotiate, monitor and manage the MPAs

SCENARIO 1

Please consider only the options given in this scenario. Compare the options below (*Option A, Option B, Current Situation*) and choose the <u>one</u> alternative that you would most prefer for Wales. Please consider the situation as if it were real and as if you would really have to pay the amount of money chosen, so please consider your budget.

- If the options below would be the only alternatives available for Wales, which one would you choose?

	Option A	Option B	Current Situation
Size of the network of MPAs	20% of coastal* waters (equivalent to 4½ times the area of Anglesey) 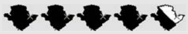	10% of coastal waters (equivalent to 2¼ times the area of Anglesey) 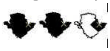	30% of coast as SAC** (equivalent to 6¾ times the area of Anglesey) 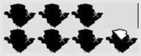
Level of Protection	Recreational and commercial fishing using non-damaging equipment to the sea floor allowed	All activities prohibited	Minimum level of protection Most activities including commercial fishing allowed
Cost to you **<u>each year</u>**	£50	£10	No additional cost to you
Which of the three options do you most prefer?	I prefer Option A ☐	I prefer Option B ☐	I prefer the Current Situation ☐

**Coastal waters*= located between the coast and 14 miles off the coast (see map)

SAC = **Special **A**rea of **C**onservation (type of MPA)

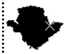 = total area of Anglesey 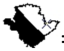 = half the area (=½ or 50%) of Anglesey

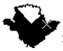 = three quarters (= ¾ or 75%) of Anglesey 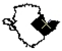 = one quarter (= ¼ or 25%)

SCENARIO 2

Consider only the options given in this scenario. Choose the one alternative that you would most prefer for Wales. Remember to consider your budget.

- If the options below would be the only alternatives available for Wales, which one would you choose?

	Option A	Option B	Current Situation
Size of the network of MPAs	20% of coastal waters (equivalent to 4½times the area of Anglesey) 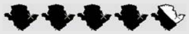	30% of coastal waters (equivalent to 6¾ times the area of Anglesey) 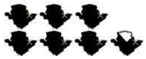	30% of coast as SAC (equivalent to 6¾ times the area of Anglesey) 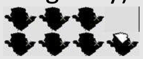
Level of Protection	Only scientific research and educational activities allowed	Non-extractive activities (i.e. sailing, diving, kayaking, wildlife watching) allowed	Minimum level of protection Most activities including commercial fishing allowed
Cost to you **each year**	£25	£5	No additional cost to you
Which of the three options do you most prefer?	I prefer Option A ☐	I prefer Option B ☐	I prefer the Current Situation ☐

SCENARIO 3

Choose the <u>one</u> alternative that you would most prefer for Wales. Remember to consider your budget.

- If the options below would be the only alternatives available for Wales, which one would you choose?

	**Option A**	**Option B**	**Current Situation**
Size of the network of MPAs	10% of coastal waters (equivalent to 2¼ times the area of Anglesey)<br>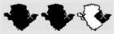	30% of coastal waters (equivalent to 6¾ times the area of Anglesey)<br>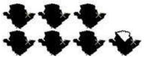	30% of coast as SAC (equivalent to 6¾ times the area of Anglesey)<br>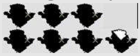
Level of Protection	Recreational and commercial fishing using non-damaging equipment to the sea floor allowed	All activities prohibited	Minimum level of protection<br>Most activities including commercial fishing allowed
Cost to you **<u>each year</u>**	£100	£25	No additional cost to you
Which of the three options do you most prefer?	I prefer Option A<br>☐	I prefer Option B<br>☐	I prefer the Current Situation<br>☐

SCENARIO 4

Choose the <u>one</u> alternative that you would most prefer for Wales. Remember to consider your budget.

- If the options below would be the only alternatives available for Wales, which one would you choose?

	Option A	Option B	Current Situation
Size of the network of MPAs	20% of coastal waters (equivalent to 4½ times the area of Anglesey) 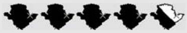	30% of coastal waters (equivalent to 6¾ times the area of Anglesey) 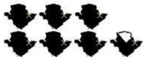	30% of coast as SAC (equivalent to 6¾ times the area of Anglesey) 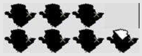
Level of Protection	Non-extractive activities (i.e. sailing, diving, kayaking, wildlife watching) allowed	Only scientific research and educational activities allowed	Minimum level of protection<br>Most activities including commercial fishing allowed
Cost to you **<u>each year</u>**	£10	£50	No additional cost to you
Which of the three options do you most prefer?	I prefer Option A ☐	I prefer Option B ☐	I prefer the Current Situation ☐

SCENARIO 5

Choose the <u>one</u> alternative that you would most prefer for Wales. Remember to consider your budget.

- If the options below would be the only alternatives available for Wales, which one would you choose?

	Option A	Option B	Current Situation
Size of the network of MPAs	20% of coastal waters (equivalent to 4½ times the area of Anglesey) 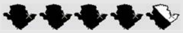	10% of coastal waters (equivalent to 2¼ times the area of Anglesey) 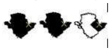	30% of coast as SAC (equivalent to 6¾ times the area of Anglesey) 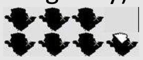
Level of Protection	All activities prohibited	Only scientific research and educational activities allowed	Minimum level of protection Most activities including commercial fishing allowed
Cost to you **<u>each year</u>**	£100	£5	No additional cost to you
Which of the three options do you most prefer?	I prefer Option A ☐	I prefer Option B ☐	I prefer the Current Situation ☐

Q11. Making choices on something that we might not know much about can cause uncertainty. How certain were you on the choices you made? (please tick one of the options)

Completely certain	Mostly certain	Somewhat certain	Slightly certain	Not at all certain
☐	☐	☐	☐	☐

Q12. If you chose the ***Current Situation*** in any of your 5 choices; why did you choose it? (please tick all responses that apply)

- ☐ I don't trust the government to be part of the programme
- ☐ I need more information to make a choice
- ☐ I support the conservation of the marine environment but cannot afford the costs
- ☐ I support the conservation of the marine environment but object to having to pay for that
- ☐ I didn't know which option was best so I stuck with the Current Situation
- ☐ No part of the sea should be closed to the public
- ☐ Options A and B restrict too many uses
- ☐ The annual costs of Options A and B are too expensive
- ☐ Enough percentage of Welsh waters are already protected
- ☐ Options A and/or B are not good enough

Q13. We would like to ask you about some of your views on the environment. Please tick the one box that resembles most closely your opinion.

	Completely True	Mostly True	Somewhat True	Slightly True	Not at all True	I am unsure
I am very interested in the environment	☐	☐	☐	☐	☐	☐
The natural environment should only be valued for what humans get out of it	☐	☐	☐	☐	☐	☐
We should protect the environment for our future generations	☐	☐	☐	☐	☐	☐
I agree with the protection of part of the Welsh sea, even if I might not use it or see it	☐	☐	☐	☐	☐	☐
Countries should cut down their economic growth in order to protect the environment	☐	☐	☐	☐	☐	☐
More money should be directed to the protection of the environment	☐	☐	☐	☐	☐	☐

7. Last section

The following section is required for data validation. This information will help us checking that all sectors of the population have been covered.

All information will remain anonymous and confidential. Information will be pooled for statistical analysis, so no individual data will be used.

Q14. What is your gender? Male ☐ Female ☐

Q15. In which year where you born? 19…..

Q16. Where do you live? (please tick as appropriate)

☐ Wales ☐ England ☐ Scotland

Q17. What is your post code? (We do not need to know your full post code please write down only the first part, i.e. if your postcode is LL57 3QR, write LL57)

____

Q18. In order that your answers to the questionnaire may be fully analysed, please could you estimate your household's gross income (i.e. before tax) for the last 12 months? (please, tick one of the boxes)

Less than £5,000	£5,000-£7,499	£7,500-£9,999	£10,000-£14,999	£15,000-£19,999	£20,000-£29,999	£30,000-£49,999	£50,000-£69,999	£70,000-£89,000	Over £90,000
☐	☐	☐	☐	☐	☐	☐	☐	☐	☐

Q19. How many people live in your household?

Adults ☐ Dependent children* ☐

* A dependent child is a person in a household aged 0 to 15 (whether or not in a family) or a person aged 16 to 18 who is a full-time student in a family with parents

Continues on the next page

→

Q20. Do you have any of these qualifications? (please all that apply)

Higher Education & professional/vocational equivalents	
Other Higher Education	
A levels, vocational level 3 & equivalents	
Trade Apprenticeships	
GCSE or equivalent	
Other qualifications (please specify)	

Q21. Which of these best describes your job? (please tick all that apply)

Agriculture/Hunting/Forestry		Real state/Renting/Business activities	
Fishing		Public administration/Defence	
Manufacturing		Education/Academia	
Electricity/Gas/Water supply		Health and Social work	
Construction		Self-employed	
Wholesale and retail trade		Full-time student	
Hotels and catering		Retired	
Transport/Storage/Communication		Unemployed	
Financial intermediation		Looking after home/family	

Other (please specify)

Thank you very much for your time and collaboration

Your help in the study is greatly appreciated

A. Ruiz
Thoday Building, Deiniol Rd
Bangor University
Bangor, Gwynedd
LL57 2UW

This work is funded jointly by the Economic and Social Research Council and the Natural Environment Research Council. It forms part of a PhD study for Ana Ruiz
